# College and Hospital Notes

**Published:** 1897-06

**Authors:** 


### College and Hospital Notes.

---

THE New York University council has finally decided the delicate question of arranging the faculty in the consolidated medical schools. The diplomacy that served to accomplish this feat to the satisfaction of all interested deserves to be recorded. It was unanimously agreed (*Boston Medical and Surgical Journal*) that the university corporation should elect the new faculty, and in order that there might be as little embarrassment as possible the members of the old ones all tendered their resignations. The corporation then furnished blanks to the professors and asked them to nominate men whom they considered most competent to fill the different chairs, and it is stated that the corporation selected nine-tenths of those who were thus recommended. While there were twenty professorships in the former medical department of the university, there are now forty-one in the new consolidated school. The following is a list of those elected :

*Professors.*—Medical ethics, Dr. John P. Munn ; pathology, Dr. J. G. Adami ; public medicine and infectious diseases, Dr. Hermann M. Biggs (Belv.) ; applied anatomy and clinical surgery, Dr. Joseph D. Bryant (Belv.) ; physiology, Dr. Austin Flint (Belv.) ; practical anatomy, Dr. Irving S. Haynes (Univ.) ; materia medica and therapeutics, Dr. Henry P. Loomis (Univ.) ; obstetrics, Dr. Wm. T. Lusk (Belv.) ; Gynecology, Dr. Wm. M. Polk (Univ.) ; theory and practice of medicine, Drs. A. Alexander Smith (Belv.) and W. Gilman Thompson (Univ.) ; surgery, Dr. Lewis A. Stimson (Univ.) ; chemistry, physics and toxicology, Dr. Rudolph A. Witthaus (Univ.) ; anatomy, Dr. George Woolsey (Univ.).

*Emeritus Professors.*—Chemistry, Dr. R. Ogden Doremus (Belv.) ; medical jurisprudence and psychological medicine, Dr. Alexander E. Macdonald (Univ.) ; otology, Dr. Chas. I. Pardee (Univ.) ; orthopedic surgery, Dr. Louis A. Sayre (Belv.) ; materia medica and therapeutics, Dr. William H. Thomson (Univ.) ; dermatology, Dr. Henry G. Piffard.

*Adjunct and Assistant Professors.*—Obstetrics, Dr. J. Clifton Edgar (Univ.) ; practical anatomy, Dr. John F. Erdmann (Belv.) ; surgery, Dr. Frederick W. Gwyer (Univ.) ; medicine, Dr. Egbert LeFevre (Univ.) ; chemistry, Dr. J. A. Mandel (Belv.) ; anatomy, Dr. George D. Stewart (Belv.) ; chemistry and physics, Dr. Ivan Sickels (Univ.).

*Clinical and Special Professors.*—Otology, Drs. Edward B. Dench (Belv.) and Gorham Bacon ; diseases of the nose and throat, Drs. F. H. Bosworth (Belv.) and Cornelius G. Coakley (Univ.) ; ophthalmology, Drs. Charles S. Bull (Univ.) and Henry D. Noyes (Belv.) ; surgery, Dr. Frederic S. Dennis (Belv.) ; diseases of the nervous system, Dr. Edward D. Fisher (Univ.) ; genito-urinary diseases, Drs. Samuel Alexander (Belv.) and P. A. Morrow (Univ.) ; midwifery, Dr. Austin Flint, Jr. (Belv.) ; dermatology, Dr. John A. Fordyce (Belv.) ; mental diseases, Dr. Carlos F. MacDonald (Belv.) ; diseases of children, Drs. Wm. P. Northrup (Belv.) and Joseph E. Winters (Univ.) ; medicine, Drs. Beverley Robinson (Belv.) and Charles E. Quimby ; lecturer on orthopedic surgery, Dr. Reginald H. Sayre.

In the above list there appear the names of but two who did not formerly occupy positions in either Bellevue or the University Medical School—namely, Drs. John P. Munn and J. G. Adami. Dr. Munn is the only member of the faculty who is also a member of the council of the university. Dr. Adami is now professor of pathology in McGill University, Montreal, and has an international reputation. He is a graduate of Cambridge University, where he formerly held an assistant professorship.

The university council held a special meeting Wednesday, May 26th, with eighteen members present, and completed the roll of professors of the consolidated University-Bellevue Hospital College by the election to full professorships in the governing faculty of Dr. E. G. Janeway, in medicine, and Dr. Frederic S. Dennis, in surgery. The roll of the governing faculty now numbers twenty-one members, while the clinical, emeritus and assistant professors are twenty-five.

---

The Riverside hospital, at 306 Lafayette avenue, Buffalo, has recently enlarged its capacity by taking the adjoining house, which increases its facilities for accommodating patients nearly 50 per cent.

Dr. Carro Julia Cummings (Buffalo University, '97,) has been appointed assistant house physician. Miss Falkner, as matron, takes charge of the hospital housekeeping, and Mrs. Margaret Johnston has been appointed superintendent of nurses. The Journal extends its congratulations to the hospital on its progress.

